# Derivation of a high-resolution CT-based, semi-automated radiographic score in tuberculosis and its relationship to bacillary load and antitubercular therapy

**DOI:** 10.1183/13993003.00600-2023

**Published:** 2023-09-07

**Authors:** Catherine Riou, Elsa du Bruyn, Grace Hyun J. Kim, Irene da Costa, Jihey Lee, Alan Sher, Robert J. Wilkinson, Brian W. Allwood, Jonathan Goldin

**Affiliations:** 1 Wellcome Centre for Infectious Disease Research in Africa and Institute of Infectious Disease and Molecular Medicine, University of Cape Town, Observatory, Cape Town, South Africa; 2 Division of Medical Virology, Department of Pathology, University of Cape Town, Observatory, Cape Town, South Africa; 3 Department of Radiology, David Geffen School of Medicine, University of California, Los Angeles, Los Angeles, CA, USA; 4 UCLA Center for Computer Vision and Imaging Biomarkers, Los Angeles, CA, USA; 5 Immunobiology Section, Laboratory of Parasitic Diseases, National Institute of Allergy and Infectious Diseases, National Institutes of Health, Bethesda, MD, USA; 6 Department of Medicine, University of Cape Town, Observatory, Cape Town, South Africa; 7 Department of Infectious Diseases, Imperial College London, W12 0NN, UK; 8 The Francis Crick Institute, London, NW1 1AT, UK; 9 Division of Pulmonology, Department of Medicine, Stellenbosch University and Tygerberg Hospital, Cape Town, South Africa

## To the Editor

Efforts to curb the TB pandemic remain hindered by the lack of objective measures to quantify disease severity and track treatment success that are valid in both HIV-1-infected and -uninfected TB patients. Ralph *et al* developed a promising radiographic scoring system [1], with baseline scores being predictive of sputum smear conversion at two months, but it is reliant on skilled readers and has not been systematically validated in predominantly HIV infected study populations of variant CD4 counts. Superior to conventional chest radiography, High-resolution computed tomography (HRCT) is a highly sensitive tool to track endobronchial TB disease extent [2]. Although recent studies have made progress in development of semi-automated methods for TB diagnosis and quantification of disease severity on chest CT [3, 4], none have assessed how these radiographic scoring systems relate to mycobacterial burden, the immune response to TB and whether they can reliably quantify the effect of antitubercular therapy (ATT) on disease affected lung. Sputum culture conversion at two months remains the gold standard measure of efficacy in trials of new TB drugs and regimens [5]. However, there is renewed interest in non-sputum-based approaches for treatment monitoring as sputum TB culture is time-consuming (negative results may take up to 42 days in liquid culture) and does not yield results in cases of culture contamination.

In the present study, we derived a computer-assisted, semi-automated quantitative radiographic scoring system (TB-CAD) applied to chest HRCT. Briefly, HRCTs underwent quantitative analysis by applying texture-based computer-aided diagnosis (CAD) at the Center for Computer Vision and Imaging Biomarkers (University of California). A computer assisted, semi-automated quantitative radiographic score of TB disease extent, called TB-CAD, was developed by modification of a previously developed algorithm [6] to detect and quantify areas of abnormality including, cavitation, consolidation, nodules, scarring and airway disease. The algorithm was run after a segmentation algorithm isolated the lung parenchyma and the TB-CAD score was calculated as the percentage of pixels of TB related abnormality present within the lungs on chest HRCT of patients with pulmonary TB and healthy controls. All participants were adults and recruited at the Site B Clinic, Khayelitsha, Cape Town, South Africa and provided written informed consent [7]. The study was approved by the UCT Human Research Ethics Committee (HREC:050/2015).

**Figure 1:**
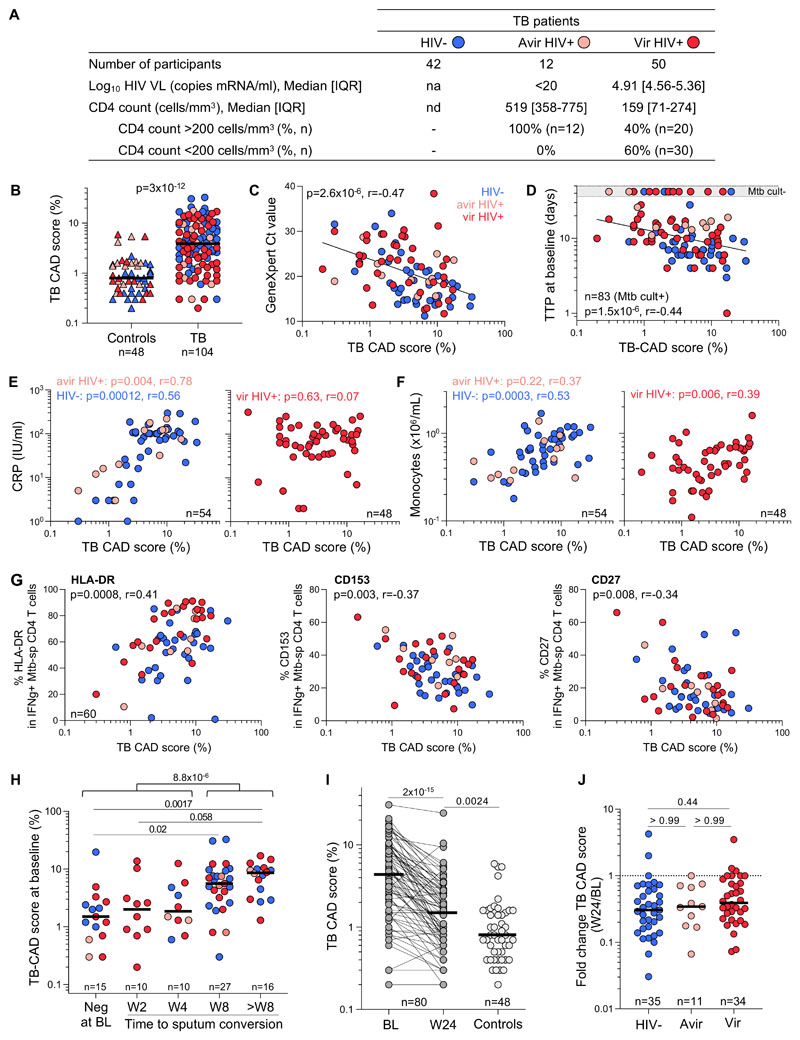
TB-CAD score relationship with TB disease activity and treatment response in HIV-uninfected and HIV-infected participants. **A)** Clinical characteristics of TB participants grouped according to their HIV status (HIV-, aviremic HIV+ and viremic HIV+).**B)** TB-CAD score in heathy controls (n=48) and pulmonary TB patients (n=104) at baseline. Bars represent medians. Statistical comparison was performed using the Mann-Whitney test. **C-G)** Relationship between TB-CAD score and Xpert Ct value (C), time to Mtb culture positivity, TTP (D), plasma CRP (E), blood monocyte absolute count (F), and the expression of HLA-DR, CD153 and CD27 on IFN-g producing Mtb-specific CD4+ T cells (G). Correlations were tested by a two-tailed non-parametric Spearman rank test. **H)** Relationship between TB-CAD score and time to Mtb culture conversion. Bars represent median. Statistical comparisons were defined using a Kruskal-Wallis test, adjusted for multiple comparisons (Dunn’s test).**I)** Evolution of the TB-CAD score between baseline and 24-week post-ATT initiation in pulmonary TB patients (n=80). Bars represent medians. Statistical comparison was performed using the paired Wilcoxon ranked test. **J)** Fold change in the TB-CAD score between baseline and 24-week post-ATT initiation in patients grouped based on their HIV status. Bars represent medians. Statistical comparisons were defined using a Kruskal-Wallis test, adjusted for multiple comparisons (Dunn’s test).

This study included 104 participants with newly diagnosed, drug-sensitive TB who tested sputum Xpert MTB/RIF (Cepheid) positive and underwent HRCT within 7 days of initiating ATT. 59.6% were HIV positive (n=62): 12 participants being aviremic and 50 having a detectable HIV-1 viral load. Clinical characteristics of TB patients are presented in Fig 1A. Eighty participants underwent repeat HRCT after completing antitubercular therapy (ATT). A group of 48 healthy controls (i.e., asymptomatic, IFN-γ release assay and sputum Xpert MTB/RIF negative and no previous TB) also underwent HRCT. 59.6% of the control participants were HIV positive (n=28), with 13 being aviremic (median CD4 count: 460 cell/mm^3^) and 15 being viremic (median: 3.83 Log_10_ mRNA copies/ml, IQR: 1.91-4.29) and a median CD4 count of 364 cells/mm^3^. The median age was comparable between the TB and control groups (36 vs 37 years old, respectively).

We examined the relationship between the TB-CAD score and TB disease activity, soluble inflammatory markers and the *Mycobacterium tuberculosis* (Mtb)-specific CD4 T cell profile in blood using flow cytometry [7], in a subset of the TB participants (n=60).The TB-CAD score was significantly higher in the pulmonary TB group at baseline compared to the healthy controls (median: 3.9 vs 0.8, p<0.0001, Fig. 1B). Moreover, when HIV- infected TB participants were grouped according to their absolute CD4 count (>200 or <200 cells/mm^3^), the TB-CAD score was significantly elevated in both groups compared to the control group (p=0.0022 and p=0.0024, respectively). Of note, about a quarter of TB patients exhibited a TB-CAD score comparable to controls (<2), this sub-group was mostly constituted of HIV-infected participants (80%) and showed a trend towards higher proportion of negative Mtb culture compared to those with a TB-CAD score >2. This supports the observation that HIV/TB co-infection often presents with limited lung involvement, indicating a paucibacillary nature [8, 9].

There was a significant inverse correlation between TB-CAD score and sputum Xpert MTB/RIF cycle threshold (Ct) value at baseline (p=2.6x10^-6^, r=-0.47), as well as sputum culture time to positivity (p=1.5x10^-6^, r=-0.44), irrespective of HIV-1 status (Fig. 1C and D). Additionally, the TB-CAD score was significantly higher in TB participants with 3+ AFB smear compared those who were smear negative (median: 7 vs 2.85, p=0.0002, data not shown). The TB-CAD score correlated with plasma C-Reactive Protein (CRP) levels at baseline in the HIV-1-uninfected and aviremic HIV-1 infected TB groups (p=0.0001, r=0.56 and p=0.004, r=0.78) (Fig. 1E). However, this correlation was absent in the viremic HIV-1-infected group (p=0.63), possibly due to HIV-associated systemic inflammation. TB-CAD scores correlated with blood monocyte counts of HIV-1 uninfected and HIV-1 infected participants (p=0.0003, r=0.53 and p=0.006, r=0.39) (Fig. 1F). As the monocyte to lymphocyte ratio (MLR) holds promise as TB biomarker [10], we also compared the relationship between sputum Mtb load and MLR or TB-CAD. We indeed found a significant correlation between MLR and Xpert Ct values (p=0.0014, r=-0.32). However, the TB-CAD showed a stronger correlation with Xpert Ct values (p=2.6x10^-6^, r=-0.47), thus offering added advantage of more closely corresponding to Mtb load.

Our group and others have shown that the activation, memory differentiation and functional profile of Mtb-specific CD4 T cells relates TB disease activity [7, 11, 12]. We found a positive association between the TB-CAD score and the expression of the activation marker HLA-DR on Mtb-specific CD4 T cells (p=0.0008, r=0.41), while moderate negative associations were observed with the expression of the memory marker CD27 and the TNF super family member CD153 on Mtb-specific CD4 T cells (p=0.008, r=-0.34 and p=0.003, r=-0.37) (Fig. 1G).

We compared TB-CAD scores to the earliest timepoint where a negative sputum culture result was registered (n=78). Those who were culture negative at baseline or at week 2 or 4 of ATT had significantly lower TB-CAD scores at baseline compared to those who only culture converted at or after week 8 (p=8.8x10^-6^, Fig. 1H). A subset of 80 eligible participants underwent repeat HRCT with TB-CAD scoring after ATT completion (Fig. 1I). TB-CAD scores significantly decreased after ATT in most participants (median: 4.65 vs 1.55, p=2x10^-15^).In five participants, the TB-CAD score increased but none of them experienced treatment failure or relapse during the 52-week follow-up period. While poor treatment adherence was not suspected, this cannot fully be excluded as pill counts or isoniazid urine testing were not conducted. However, TB-CAD scores only partially normalized, as TB-CAD scores post-ATT remained significantly higher than those of the control group (p=0.0024, Fig. 1I). The median fold change of TB-CAD scores between baseline and post-ATT was comparable, regardless of HIV status (Fig. 1J).

Overall, we show that the TB-CAD score correlated with TB bacillary load, as evidenced by its inverse correlation to both sputum Xpert Ct value and culture time to positivity, whilst significantly declining post-ATT, regardless of HIV status. Furthermore, TB-CAD scores correlated with blood monocyte count and CRP, both of which are markers of systemic inflammation, usually elevated in TB, and the latter poorly prognostic [13, 14].

Focusing on more specific readouts of TB disease activity, we evaluated the Mtb-specific CD4+ T-cell profile in relation to the TB-CAD score. CD153 has been implicated as marker of protection, with the proportion of Mtb-specific CD4 T cells expressing CD153 significantly lower in active compared to latent TB [11]. It is thus noteworthy that higher TB-CAD scores were associated with a more differentiated (CD27^low^), highly activated (HLA-DR^high^) Mtb-specific CD4+ T-cell profile with low CD153 expression. While these correlations are moderate, it is reassuring that Mtb-specific immune responses known to associate with TB disease activity [7, 11] also relate to radiographic disease extent. The main limitations of our study include the absence of an IGRA positive control group, and the significant differences in ART uptake between the HIV-1 infected study groups. However, this does not detract from our main finding that the TB-CAD score offers an objective, quantitative readout of TB disease extent in the lung, regardless of HIV-1 status. This is significant as chest radiographs, commonly used for diagnostic and treatment monitoring purposes in TB, can frequently be normal or display non-specific features in TB-HIV coinfected individuals, specifically in those with low CD4 counts, who in turn represent those at highest risk of TB related mortality [8, 9]. Furthermore, these individuals are often sputum smear negative owing to low bacillary load, leading to treatment monitoring challenges [15]. This highlights the importance of investigating non-sputum-based technologies. Further studies are needed to validate our findings in an independent cohort, including participants with CD4 counts <50 cells/mm^3^ and determine whether TB-CAD scores at earlier timepoints during ATT can predict outcome. We envisage that the TB-CAD score has potential utility in the clinical trial setting where real time, quantitative data reflecting efficacy of experimental drugs in reducing mycobacterial burden and inflammation, in both HIV-1-infected and - uninfected participants, is critical.
